# Histomorphometric and immunohistochemical assessment of treated dentin matrix delivered by platelet-rich fibrin for socket preservation in rabbit model

**DOI:** 10.1186/s12903-025-05569-3

**Published:** 2025-02-12

**Authors:** Eman M. Sedek, Nesma Mohamed Khalil, Mohamed M. Abdul-Monem

**Affiliations:** 1 Department of Dental Biomaterials, Faculty of Dentistry, Alexandria University, Alexandria, Egypt; 2 Department of Oral Biology, Faculty of Dentistry, Alexandria University, Alexandria, 21525 Egypt

**Keywords:** Treated dentin matrix, Nanobone, Platelet-rich fibrin, Rabbits, Tooth extraction, Socket preservation

## Abstract

**Objectives:**

This study aimed to test treated dentine matrix (TDM) with platelet-rich fibrin (PRF) for socket preservation, following tooth extraction compared to synthetic grafting material Nanobone (NB) with PRF.

**Materials and methods:**

**S**tudy was conducted on New Zealand rabbits (*n* = 40). Bilateral first lower premolar extraction was performed, with one side left empty and the other side filled with (PRF) in one group (*n* = 20). In the other group (*n* = 20), one side was filled with TDM/PRF while the other side was filled with NB/ PRF. After one and three months, rabbits were euthanized, and the socket area was examined using haematoxylin and eosin (H&E) and Goldner Masson trichrome stains. One-way ANOVA and post-hoc tests were used for histomorphometric analysis. Immunohistochemical analysis of osteopontin was carried out.

**Results:**

Histological analysis of NB/PRF and TDM/PRF groups showed a higher level of new bone formation in comparison to the control and PRF groups. Histomorphometric analysis revealed a significant increase in new bone formation in the TDM/PRF group compared to the NB/PRF group after one and three months (*p* = 0.042 and *p* < 0.001), respectively. There were no significant differences in the percentages of unmineralized bone between the TDM/PRF and NB/PRF groups at both intervals (*p* = 0.375 and 0.352, respectively). Regarding immunohistochemistry, NB/PRF showed the highest osteopontin immune expression followed by TDM/PRF. No significant differences were detected between both groups at both intervals (*p* = 0.234 & 0.607 respectively).

**Conclusions:**

TDM/PRF showed the ability to form new bone in extraction sockets in rabbits.

**Clinical relevance:**

TDM/PRF can be used as an alveolar bone grafting material for socket preservation.

**Supplementary Information:**

The online version contains supplementary material available at 10.1186/s12903-025-05569-3.

## Introduction

Following extraction of teeth, resorption of bone becomes apparent, characterized by the atrophy of the alveolar ridge. In general, an overall clinically significant reduction in bone height of around 2–5 mm can be vertically observed within the initial 6-month period [1, 2]. After a period of 12 months, it is observed that alveolar ridge may show a reduction in width of approximately 50%. This phenomenon has significant implications in the context of dental implants, suggesting that the successful insertion of an implant in an inadequate bone foundation may be challenging.

Various techniques have been proposed to address the issue of bone atrophy and its prevention, known as socket preservation [3, 4]. The application of deproteinized bovine bone for preserving extraction sockets is widely recognized in clinical practice and has been the subject of investigation in multiple research studies. In addition, clinical procedures such as the socket-shield technique are carried out [5, 6]. In the field of dentistry, the main source utilized for bone grafting consists of allogeneic bone and synthetic mineral materials. Fresh autogenous bone graft remains widely regarded as the most superior option due to its display of bioactive cell-instructive matrix characteristics, along with being non-immunogenic and non-pathogenic. Nevertheless, the limitations linked to this approach encompass the requirement for bone harvesting and the possible adverse health effects that might result from the process [7].

The advancement in material technology has led to the development of treated dentin matrix (TDM), also referred to as autogenous dentin particles, which exhibits favorable biocompatibility, absence of immune response and mitigates the risk of transmission of diseases [8–11]. Furthermore, dentin’s chemical characteristics display a strong correlation with bone tissue and have exhibited effective osseous regeneration in both animal and human experimental settings [8, 9].

Although TDM is present as a blocky solid substance, it poses challenges in terms of manipulation and retention post-implantation. Numerous research endeavors have made efforts to develop TDM formulations that exhibit enhanced manipulability [12]. Hence, the incorporation of certain additives like autologous platelet-rich fibrin (PRF) as a vehicle for TDM might offer improved handling characteristics to the material while preserving its biological activity.

The utilization of PRF is a biotechnological method that has been scientifically validated to enhance and quicken the healing of bones and wounds, especially within the context of orthopedic surgical literature [13, 14]. PRF can be derived from plasma using a centrifugation technique, which commences with whole blood. The capacity of PRF to rejuvenate hard and soft tissues by means of angiogenesis and growth factors, in conjunction with the ease with which the surgeon can obtain this substance, renders it a promising material for socket preservation [15].

Several studies have reported success using both TDM and PRF separately [16–18]. Hence, the primary objective of this study was to test the recuperative dynamics of combining TDM with PRF for socket preservation, post tooth extraction when compared to synthetic bone grafting substance (NB) along with PRF. Furthermore, characterization of the prepared grafts will be done to help interpret our results. The null hypothesis is that TDM/PRF and NB/PRF would show no significant differences in bone formation.

## Materials and methods

The methodology of this study was carried out in accordance with ARRIVE (Animal research: Reporting in vivo experiments) guidelines [19].

### Fabrication of TDM

Freshly extracted lower first premolars of New Zealand white rabbits were used to obtain TDM. The coronal part and pulp tissues were removed, then cementum was eliminated, and the roots were perforated to allow the perfusion of ethylenediaminetetraacetic acid (EDTA) solution, using 17%, 10%, and 5% EDTA for 3–4 min, 5 min, and 2 min, respectively. The use of different EDTA concentrations was done to partially demineralize TDM and efficiently preserve proteins and functional factors as previously investigated [20–23], as partial demineralization of TDM was a key step in the current study [21, 24]. Teeth were placed in a container containing 70% ethanol for a duration of 10 min to eliminate soft tissue, bacteria, and smear layer. The resultant TDM was then ground using a sterile powder grinder (Laymax grinder, Guangdong, China) for 3 to 10 s. The powder was passed through a series of sieves (Gaofu, Xinxiang, China) to obtain 500 μm particles (range 350–500 μm).

### PRF and scaffolds preparation

Rabbit blood (10 ml) was drawn through the auric vein using a syringe with a gauge needle [25], and then the blood was discharged into a test tube without any anticoagulants. The tube was placed in a PRF centrifuge (PRF DUO, Ostralos, New Zealand) and a test tube containing 10 ml of water was placed on the opposite side to achieve balance during centrifugation. The speed was set at 3000 rpm for a period of 10 min. The sample was separated into three layers following centrifugation: the top layer was supernatant, the middle layer was PRF gel, and the bottom layer was red blood cell (RBC) debris. The middle layer was retrieved using sterile tweezers, and the RBC layer was removed from the PRF gel. Consequently, a flexible PRF gel was produced [18, 26, 27].

**Fig. 1:**
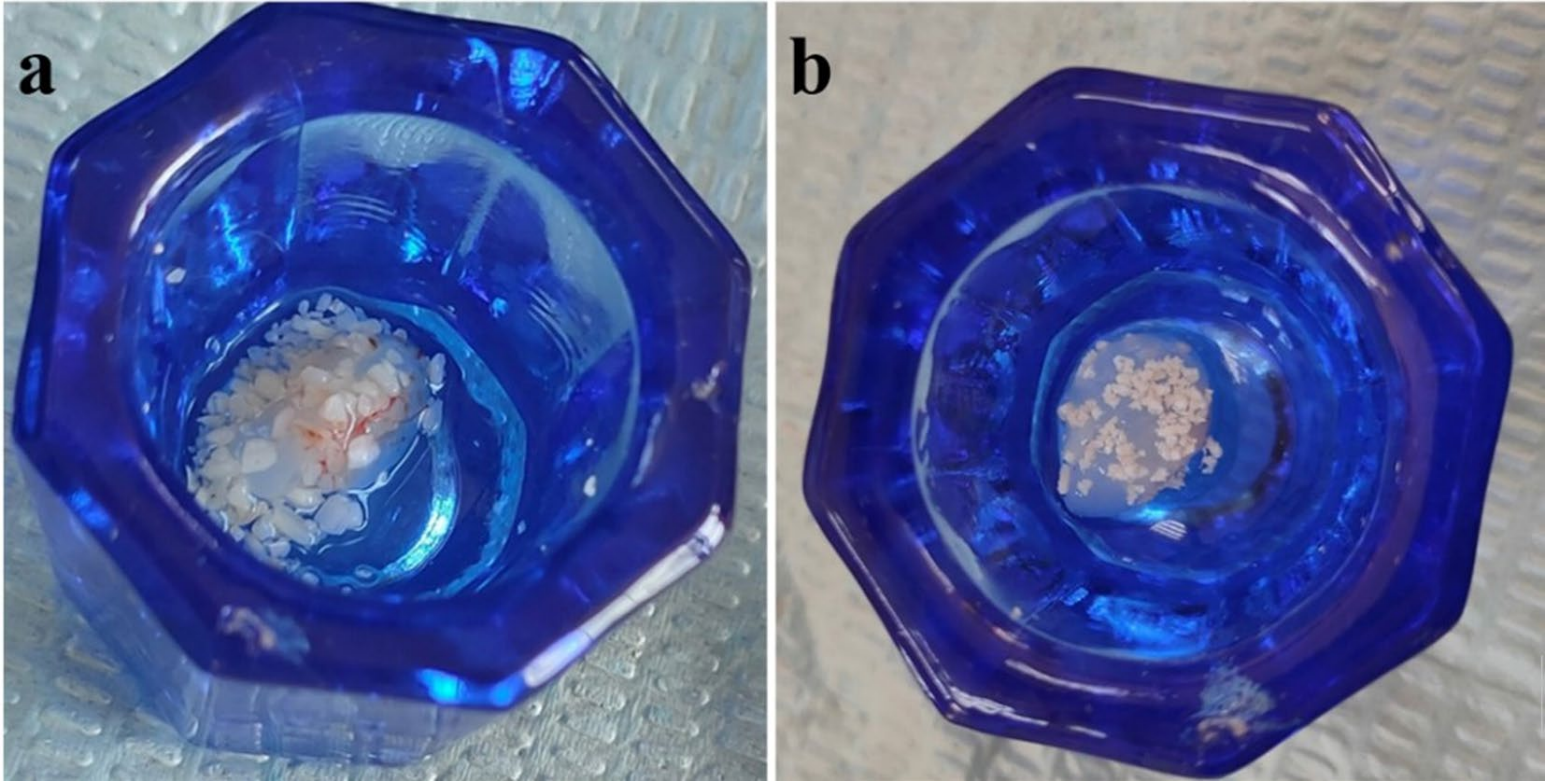
TDM/PRF scaffold (**a**), and NB/PRF (**b**)

Under aseptic conditions, sterile TDM and NB (600 μm) were mixed with PRF by a ratio of 1:1 (Fig. 1a and b), respectively. This ratio was selected based on preliminary trials to maximize TDM and NB content to obtain the effective amount of fillers [28, 29].

### Characterization of the prepared scaffolds

Fourier Transform Infrared Spectroscopy (FTIR) spectra were obtained through the utilization of an FTIR spectrophotometer (Perkin-Elmer, Massachusetts, USA) with the aim of identifying the functional groups present in TDM/PRF and NB/PRF within the spectral range of 400–4000 cm^− 1^ [17, 23]. The surface morphology and porosity of gold-coated specimens of lyophilized TDM/PRF and NB/PRF scaffolds were examined using a scanning electron microscope (SEM), (JEOL, Tokyo, Japan) [28].

### Sample size calculation

Sample size was estimated assuming a 5% alpha error and 80% study power. The mean (SD) percentage of newly formed bone after 3 months was 80.54% (13.28) and 81.7% for PRF and NB, respectively [30, 31]. According to Nam et al. [32], the mean percent of newly formed bone in the dentin matrix group was 29.53% (7.65), 36.31% (9.8), and 52.27% (4.44) at 2 weeks, 4 weeks, and 8 weeks, respectively. Based on the previous values, it was estimated that after 3 months, 60% (7.30) of bone will be formed. The determination of the minimum sample size per group in a single-time interval was achieved through the comparison of independent means utilizing the F test and a pooled standard deviation of 8.79. This initial calculation resulted in 8 defects per group, a figure which was subsequently adjusted to 10 defects to account for potential processing errors or subjects experiencing mortality during the study. Consequently, the total sample size was computed using the formula: Total sample = number per group x number of groups x number of time intervals = 10 × 4 × 2 = 80 defects. Notably, this corresponded to a total of 40 rabbits, with both sides of each rabbit being utilized in the study. The methodology employed for determining the sample size was in accordance with Rosner’s approach [33], calculated by G*Power 3.1.9.7.

### Study setting

The present study was approved by the Institutional Ethical Committee, Faculty of Dentistry, Alexandria University (IRB No. 00010556-IORG No. 0008839) (0502-09/2022). The authors followed all institutional and international guidelines for animal care and use during this study. The Animal Research: Reporting in Vivo Experiments guidelines (ARRIVE) were also followed. Rabbits were obtained from the animal house of the Medical Research Institute, Alexandria University. Rabbits were housed in polypropylene enclosures within the experimental animal facility, maintaining consistent environmental conditions such as 12-hour light-dark cycles, a temperature of 24 ± 2 °C, and ad libitum access to water and a commercial diet.

### Study design and grouping

**Fig. 2:**
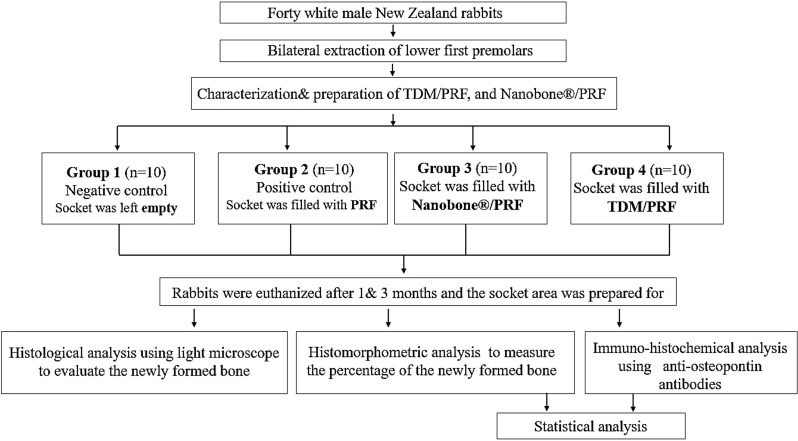
The flow chart of study design

The experiment was conducted on the mandibular first premolars of rabbits following a 1:1 allocation ratio (split-mouth design), specifically targeting two lower premolars - one situated on the right side and the other on the left side of each rabbit. All specific sites underwent a healing process lasting either one or three months. During each designated healing duration, ten rabbits were subjected to socket preservation materials, utilizing TDM/PRF on one side and NB/PRF on the opposite side. Simultaneously, additional ten rabbits were designated as control subjects, with one side treated with a blood clot while the other side was treated with PRF only (Fig. 2). Within this randomized controlled experimental design, the animals were distributed randomly and uniformly across four distinct groups. Every rabbit was assigned a unique number and affixed to the ear for randomized allocation based on a pre-generated table through a computer-derived random sequence.

### Inclusion and exclusion criteria

A total of forty male New Zealand white rabbits, weighing between 3.5 and 4.5 kg and aged approximately one year, were enlisted as experimental subjects, with twenty rabbits allocated for each study duration (one and three months). Female rabbits were specifically excluded due to cyclic hormonal variations and the potential for pregnancy throughout the experimental timeline.

### Surgical procedures

All surgical procedures adhered to a standardized anesthesia protocol. Specifically, the premedication consisted of 25 mg/kg ketamine and 5 mg/kg xylazine delivered through the auricular vein. After orotracheal intubation, anesthesia was sustained throughout the procedure by inhaling a blend of oxygen and isoflurane (1–1.5 vol%). Analgesia (4 mg/kg carprofen) was administered before and after the surgery intravenously and continued for 5 days intramuscularly [24]. Extraction of teeth was performed delicately without elevating a flap or compromising the vestibular or lingual bone structures.

### Histological analysis

The animals were euthanized through the administration of an excessive amount of pentobarbitone at a dosage of 600 mg/kg after a healing period lasting either one or three months. The dissection of the jaws was meticulously conducted to retrieve tissue blocks encompassing the corresponding experimental regions. After fixation in 10% neutral buffered formalin, the specimens underwent a washing process followed by decalcification in 5% formic acid. The samples were dehydrated by placing in increasing alcohol concentrations, then clarification in xylene, and finally permeation and embedding in paraffin wax. Sections (4–5 mm) in thickness were laterally sliced from the paraffin blocks and subjected to staining with hematoxylin and eosin (H & E) for comprehensive evaluation of the newly synthesized bone, which was performed under a light microscope [34] and Goldner Masson trichrome stain to detect the unmineralized bone [26, 35]. To mitigate the risk of potential bias, a pair of expert examiners conducted a thorough examination of all specimens in the study, employing coded samples [36]. Inter- and intra-examiner reliability were assessed through the employment of a minimum Cohen kappa value of 0.87 being deemed satisfactory.

### Histomorphometric analysis

Image J software (Wayne Rasband, Maryland, USA) was used for histomorphometric evaluation [37]. The analysis was carried out in four serial sections for each specimen in each group, and the mean was obtained. The following parameters were calculated:

1. **New bone% (%)**: new bone was traced, and the (%) was calculated in relation to the total surface area of the field.
2. **Percentage (%) of unmineralized bone (stained red)** and its area (%) to the total surface area of the field.

### Immuno-histochemical analysis

Immunohistochemical analysis of osteopontin was carried out using anti-osteopontin antibodies (Medaysis, Livermore, CA, USA). The optical density of osteopontin immune-staining was calculated using the following equation: OD = log (maximum intensity/mean intensity), maximum intensity = 255 [38]. The analysis was carried out in four serial sections in each specimen and the mean was obtained.

### Statistical analysis

Data obtained from histomorphometric examination underwent normality assessment through the Shapiro-Wilk test. Numeric values were presented as a range encompassing the minimum and maximum values, along with the mean, standard deviation, and median. To analyze differences among the various groups under investigation, a One-way ANOVA test was conducted, followed by a Tuckey post-hoc test for pairwise comparisons. In instances where quantitative variables exhibited a normal distribution, a student t-test was employed to compare two distinct groups. The predetermined level of significance for the findings was established at (*p* = 0.05). Data was analysed using IBM SPSS software package version 20.0. (Armonk, NY: IBM Corp).

## Results

### Characterization of the prepared scaffolds

**Fig. 3:**
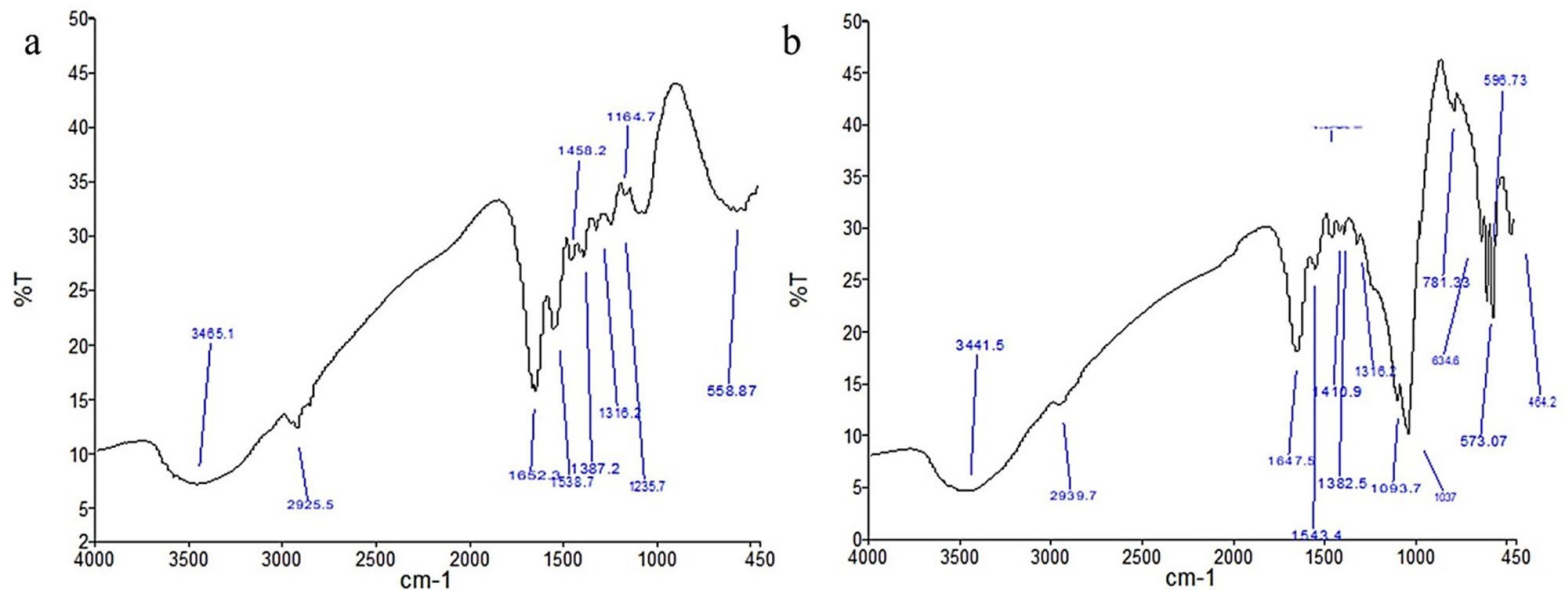
FTIR analysis of TDM/PRF (**a**), and NB/PRF (**b**)

FTIR results of TDM/PRF are shown in (Fig. 3a) and revealed characteristic peaks of phosphate groups (PO4^− 3^) at 558 cm^− 1^ and 900–1100 cm^− 1^, with the highest band at 1033 cm^− 1^. Carbonate groups (CO_3_) can be seen at 870 cm^− 1^. Peaks observed at 1652 cm^− 1^, 1538 cm^− 1^, and 1235 cm^− 1^ correspond to the presence of amide I, amide II, and amide III, representing the dentine proteins and fibrin. The peak observed at 1458 cm^− 1^is associated with the (CH_2_) functional group, while the peak identified around 3465 cm^− 1^may be linked to the existence of hydroxyl groups and the amide N–H bond.

The FTIR findings of NB/PRF are shown in (Fig. 3b) with characteristic peaks of NB manifested as peaks located at 464 cm^− 1^ and 781 cm^− 1^, which correspond to silica (Si–O–Si). The distinctive peaks of phosphate functional groups (PO4 − 3) are evident at 558 cm^− 1^ and within the range of 900–1100 cm^− 1^, with the predominant band observed at 1033 cm^− 1^. The presence of the carbonate group (CO_3_) is identifiable at 1410 cm^− 1^, while the peak detected around 3441 cm^− 1^ is associated with the existence of hydroxyl groups and the amide N–H.

**Fig. 4:**
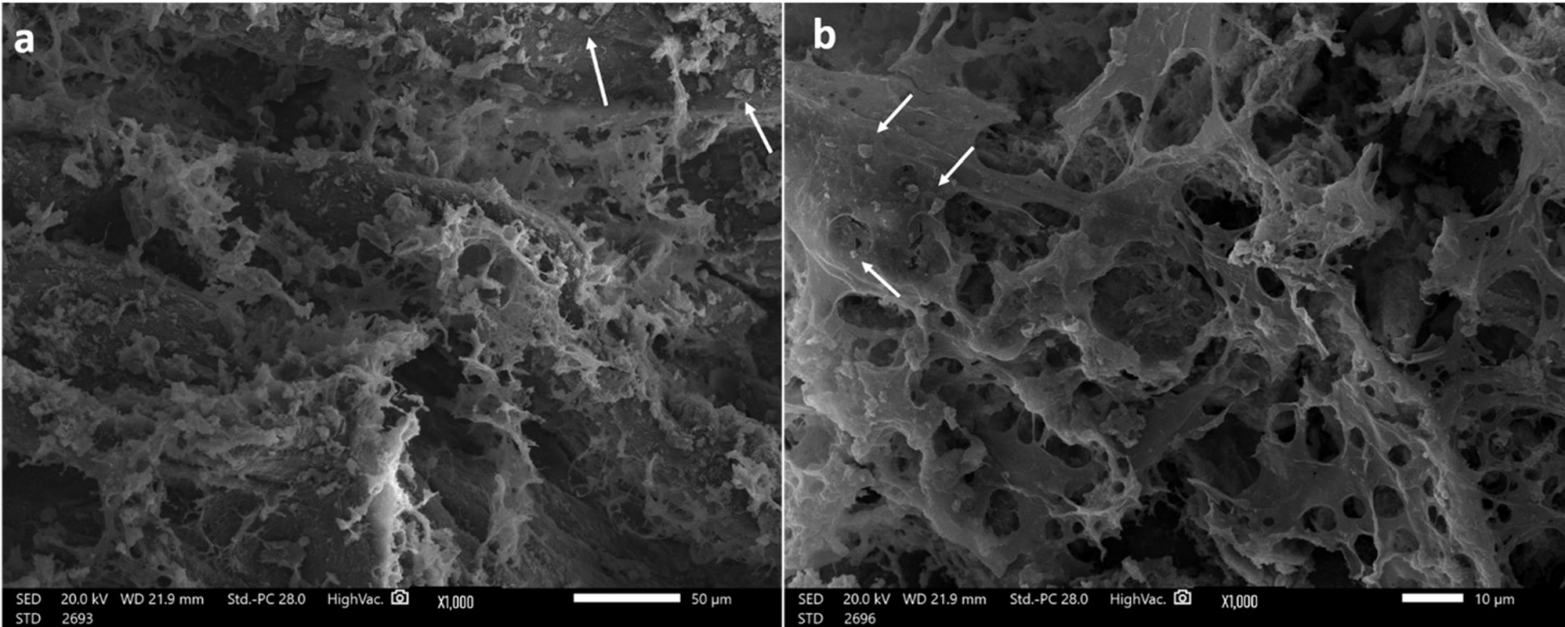
SEM images of the freeze-dried TDM/PRF(**a**) and NB/PRF (**b**) (with original magnifications ×1000 at 20 kV) showing particles of TDM and NB (arrows) attached to the PRF matrix

SEM images of freeze-dried TDM/PRF and NB/PRF revealed the PRF matrix organized in line-like structures, forming a mesh-like structure with varying degrees of porosities and leukocytes present. Particles of TDM and NB can be seen attached to the PRF matrix at (x1000) (Fig. 4).

### Histological analysis

#### One-month period

**Fig. 5:**
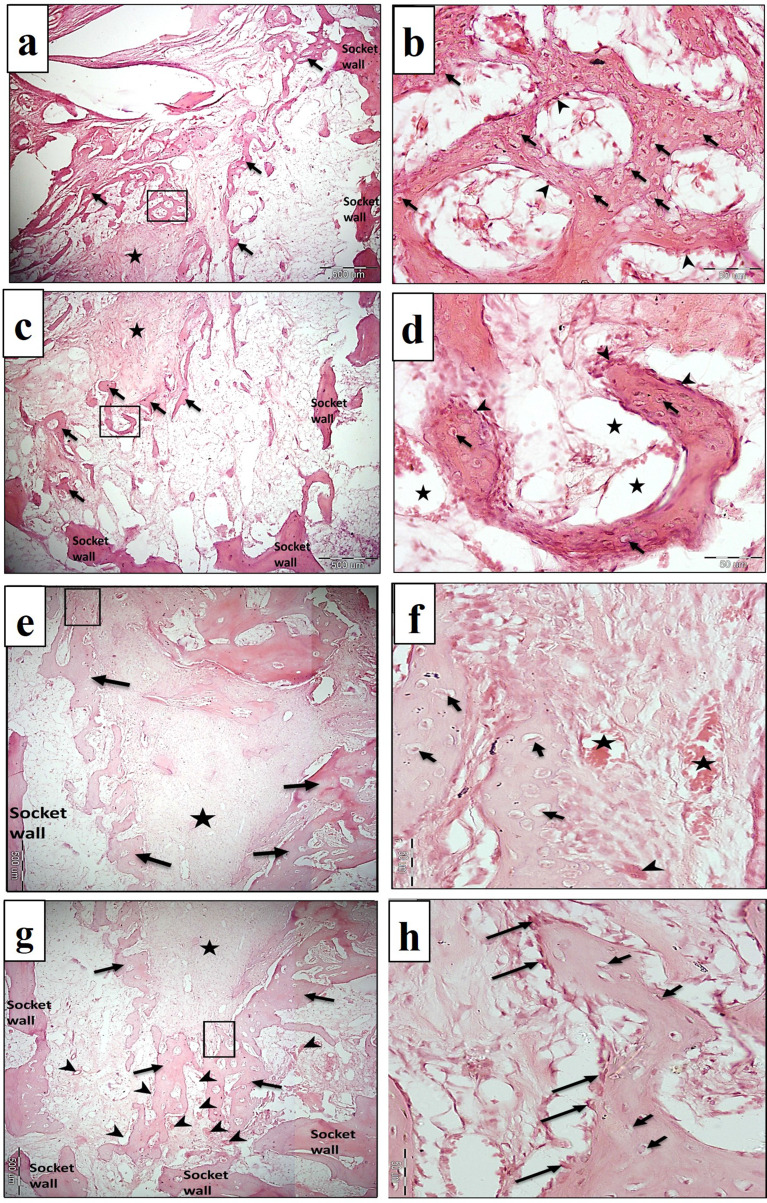
Light micrograph (LM) of control group (**a**-**d**) and PRF group (**e**-**h**) in the first month. **(5a and 5c)** show coronal and apical half of the socket of control group respectively. Scattered newly formed bony spicules are seen in the socket (arrows). The central part contains fibrous tissue (star). **(5b and 5d)** are higher magnifications of (5a and 5c) respectively. The bony spicules contain numerous, large haphazardly arranged osteocytes lacunae (arrows) and covered by voluminous osteoblasts (arrow heads). Numerous blood vessels (stars) can be seen. (H&E, 5a and 5c, x40, 5b and 5d x400). **(5e and 5 g)**: Show coronal and apical half of the socket of PRF respectively. Cancellous bone (arrows) is seen on each side of the socket wall. The central part of the defect is filled with fibrous tissue (asterisks). Note the intense vascular supply (arrow heads) in the apical part of the socket. **(5f and 5 h)** are higher magnifications of (5e and 5 g), respectively showing relatively large and regularly distributed osteocyte lacunae (short arrows), osteoclast (arrow head), numerous blood vessels (asterisks) and voluminous osteoblasts (long arrows). (H&E, 5e and 5 g x40, 5f and 5 h x400)

A light microscopic examination of specimens from the negative control group showed the formation of a few scattered spicules of immature woven bone all over the socket area. The spicules contain numerous haphazardly arranged large osteocyte lacunae and are covered by osteoblast cells. The central part of the socket contained fibrous tissue (Fig. 5a-d).

In the PRF group, more bone was formed, especially in the apical part of the socket. The bone consisted of cancellous bone, which contained regularly distributed osteocytes and was covered by plump osteoblasts, which indicates active bone formation. In some areas, the osteocyte lacunae appeared large. Intense blood supply was seen specifically in the apical part of the socket (Fig. 5e-h).

**Fig. 6:**
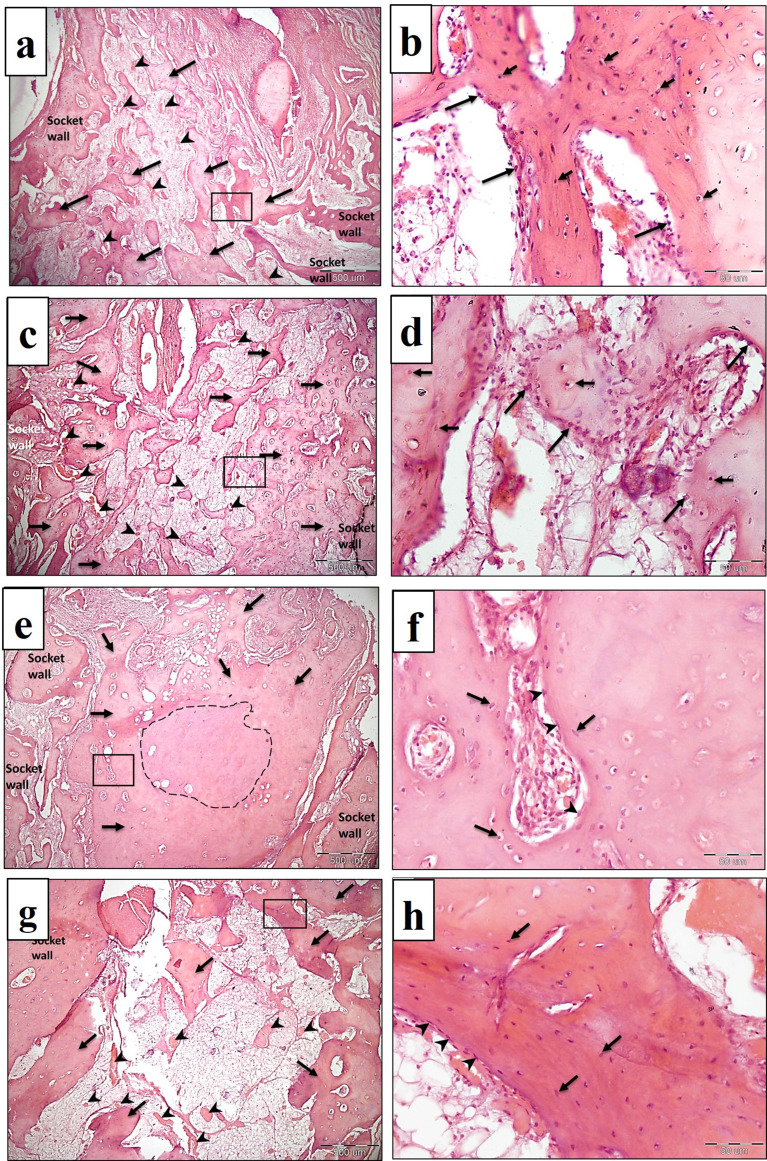
LM of NB/PRF group (**a**-**d**) and Treated dentin matrix/PRF group (**e**-**h**) in the first month. **(6a and 6c)** show coronal and apical half of the socket of NB/PRF group respectively. Many trabeculae of cancellous bone (arrows) can be seen filling the socket area. Numerous blood vessels (arrow heads) are seen in the bone marrow spaces. **(6b and 6d)** are higher magnifications of (6a and 6c) respectively. Mature bony trabeculae with regularly distributed osteocytes (short arrows) and plump osteoblasts (long arrows) are seen. (H&E, 6a and 6c x40, 6b and 6d x400). **(6e)** shows the coronal half of the socket of TDM/PRF group. Obvious bone formation (arrows) surrounds dentin remnants (dotted area). **(6 g)** shows the apical half of the socket containing cancellous bone trabeculae (arrows) and numerous blood vessels (arrow heads). **(6f and 6 h)** are higher magnifications of (6e and 6 g) respectively. The bone trabeculae contain well organized osteocytes (arrows) and osteoblasts lining its endosteal surface (arrow heads). (H&E, 6e and 6 g x40, 6f and 6 h x400)

The NB/PRF group showed the formation of more cancellous bone trabeculae all over the coronal and apical parts of the socket in comparison to the control and PRF groups. The bone trabeculae contained well-organized osteocyte lacunae and were lined by voluminous osteoblasts. Well-vascularized bone marrow tissues were noted all over the socket area (Fig. 6a-d).

The TDM/PRF group showed obvious bone formation in the coronal half of the socket surrounding remnants of the dentin matrix. In the apical half, cancellous bone trabeculae were seen, which contained regularly distributed osteocyte lacunae and were lined by osteoblasts. Numerous blood vessels were seen in the bone marrow (Fig. 6e-h).

### Three-month period

**Fig. 7:**
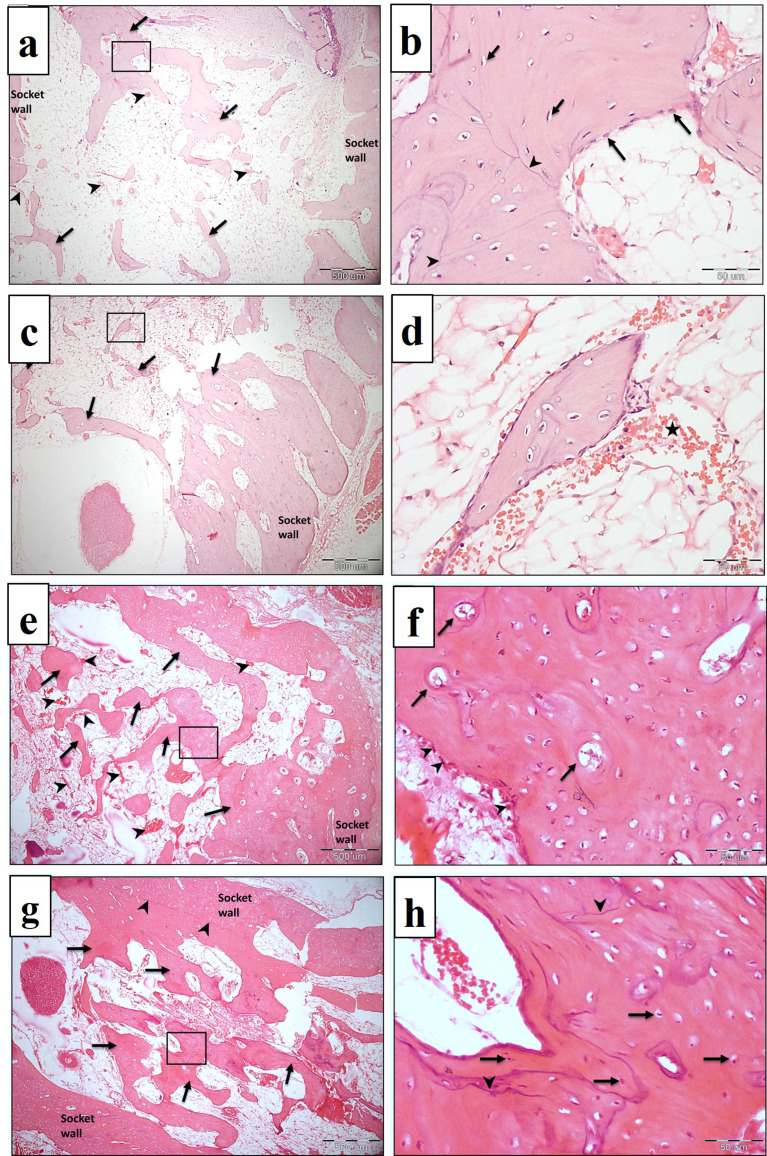
LM of control group (**a**-**d**) and PRF group (**e**-**h**) in the third month. **(7a and 7c)** show coronal and apical half of the socket of control group respectively. The socket contains scattered few trabeculae (arrows) surrounding fatty bone marrow spaces with scattered blood vessels (arrow heads). **(7b and 7d)** are higher magnifications of (7a and 7c) respectively showing the structure of bony trabeculae with osteocyte lacunae (short arrows), osteoblasts (long arrows) and surrounded by blood vessel (asterisk). Reversal lines are also seen (arrow heads). (H&E, 7a and 7c x40, 7b and 7d x400). **(7e)** the coronal half of the socket of PRF group shows the formation of numerous trabeculae of cancellous bone (arrows) surrounding well vascularized (arrow heads) bone marrow spaces. **(7f)** higher magnification showing the formation of small osteons (arrows) and plump osteoblasts (arrow heads) covering the bone surface. **(7 g)** shows the apical half of the socket which contains thick trabeculae of cancellous bone (arrows), and line of demarcation (arrow heads) is seen between old bone and newly formed bone. **(7 h)** Higher magnification showing regularly distributed osteocytes lacunae (arrows) and the presence of numerous reversal lines (arrow heads). (H&E, 7e and 7 g x40, 7f and 7 h x400)

Histological examination of specimens obtained from the negative control group revealed the formation of a few scattered trabeculae of cancellous bone surrounding fatty bone marrow spaces, which contained scattered blood vessels. Reversal lines were seen, which indicate bone remodeling (Fig. 7a-d).

In the PRF group, more cancellous bone formation was seen all over the socket area. The trabeculae were thicker compared to the control group, especially in the apical half of the socket. Small osteons were also seen, and numerous reversal lines were detected. The bone marrow tissues were well vascularized (Fig. 7e-h).

**Fig. 8:**
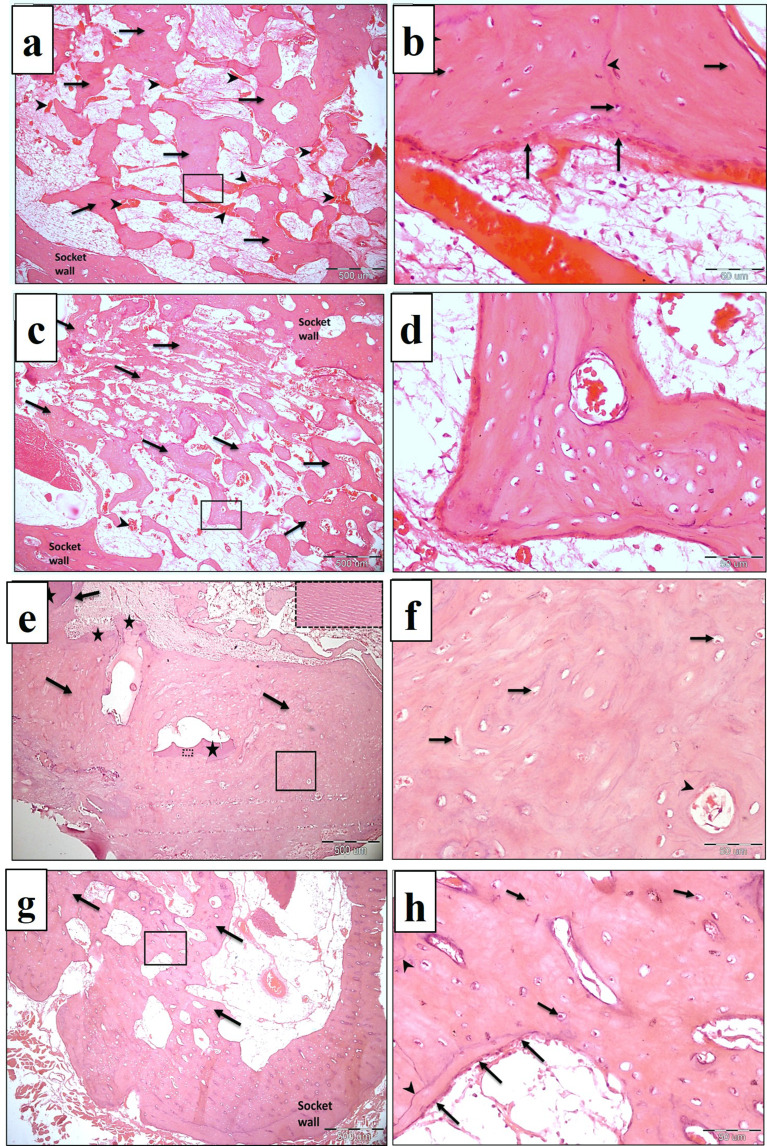
LM of (NB/PRF) group (**a**-**d**) and (TDM/PRF) group (**e**-**h**) in the third month. **(8a and 8c)** show coronal and apical half of the socket of NB/PRF respectively. Numerous cancellous bone trabeculae (arrows) are seen surrounding well vascularized bone marrow spaces (arrow heads). **(8b and 8d)** are higher magnifications of (8a and 8c) respectively showing homogenously distributed osteocytes’ lacunae (short arrows), plump osteoblasts (long arrows), and numerous reversal lines (arrow heads). (H&E, 8a and 8c x40, 8b and 8d x400). **(8e)** the coronal half of the socket of (TDM/PRF) group shows the formation of dense bone (arrows) surrounding remnants of dentin fragments (asterisks). The dotted rectangle in the upper right side is the higher magnification (x400 of remnants of dentin fragments (dotted small inset) **(8f)** higher magnification showing osteocytes (arrows) and an osteon (arrow head). **(8 g)** the apical half of the socket shows the formation of cancellous bone trabeculae (arrows). **(8 h)** higher magnification showing regularly distributed osteocytes (short arrows), osteoblasts (long arrow) lining the endosteal surface of bone and reversal lines (arrow heads) (H&E, 8e and 8 g x40, 8f and 8 h x400)

NB/PRF group showed the formation of more numerous cancellous bone trabeculae occupying the whole socket area and surrounding bone marrow tissues, which exhibited profound blood supply and reversal lines were also seen (Fig. 8a-d).

In the TDM/PRF group, more compact bone formation was seen, especially in the coronal half of the socket, surrounding remnants of the dentin matrix. In the apical half, cancellous bone trabeculae were seen, which contained regularly arranged osteocytes and reversal lines (Fig. 8e-h).

### Trichrome stained sections

**Fig. 9:**
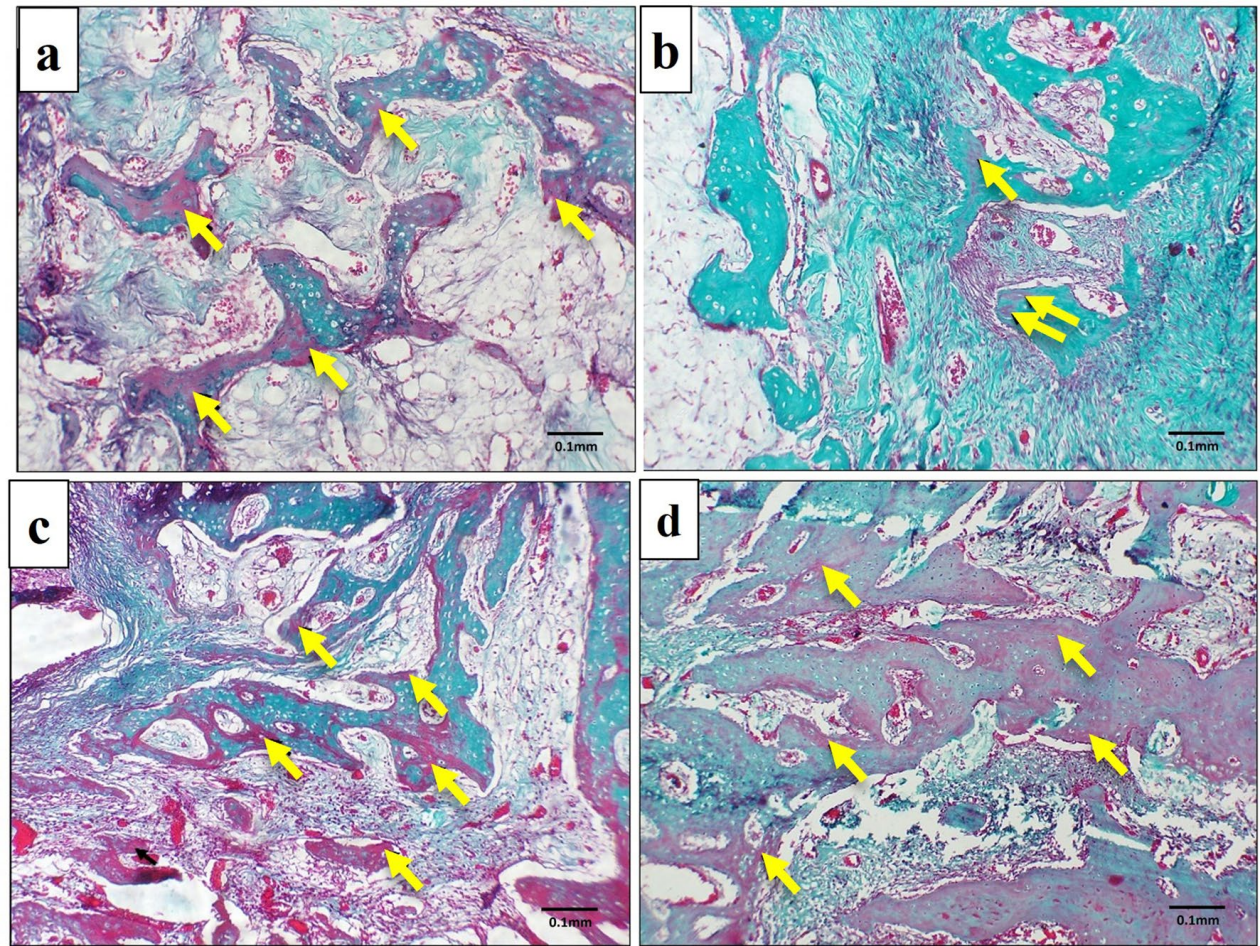
LM of the experimental groups at one month showing the unmineralized areas of bone (arrows). **a**: Control group, **b**: PRF group, **c**: NB/PRF group, **d**: TDM/PRF group. Masson Goldner trichrome, x100

**Fig. 10:**
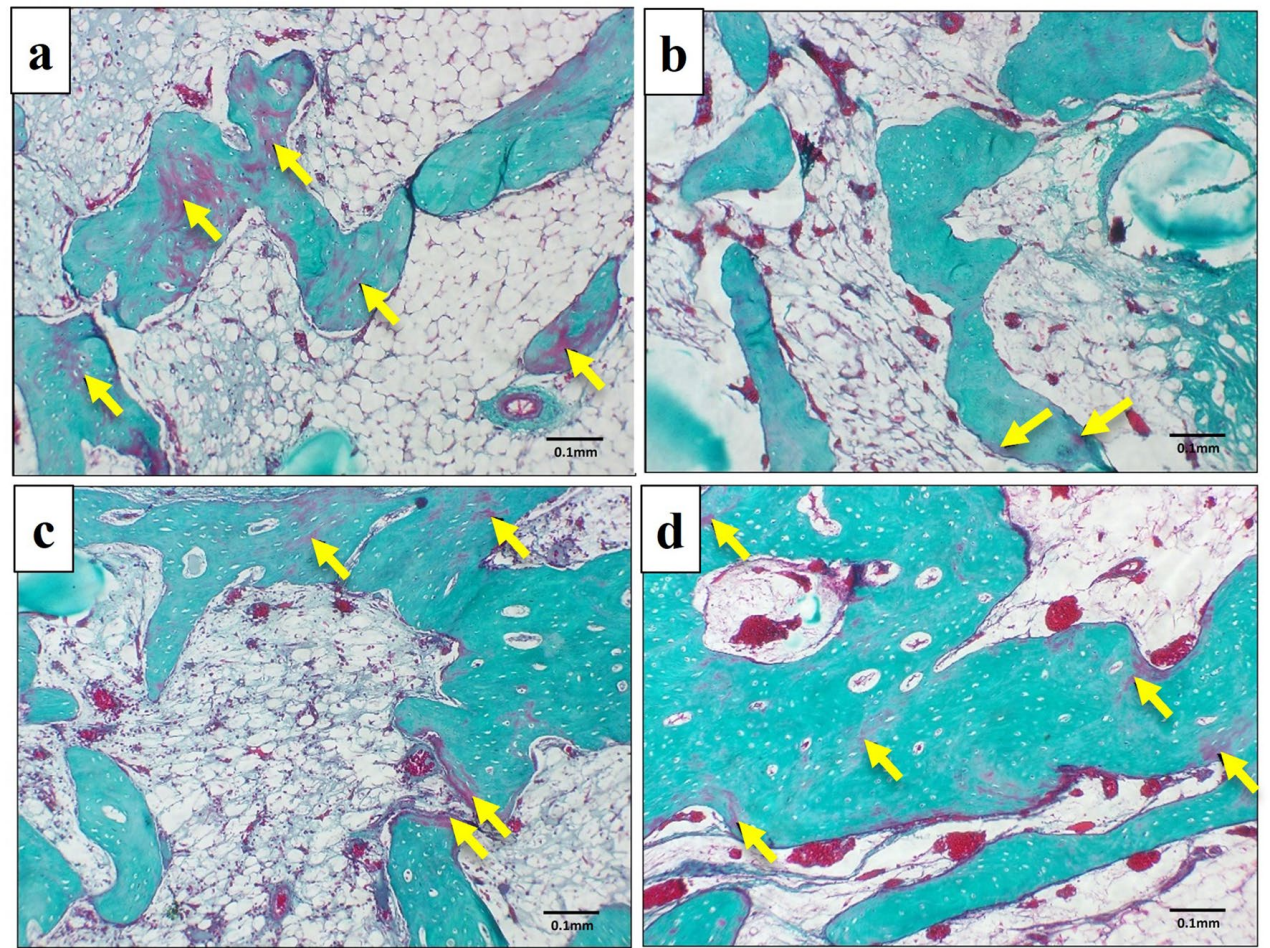
LM of the experimental groups in the third month showing homogenous mineralization of bone matrix (stained green) with few scattered areas of unmineralized bone (arrows). **a**: Control group, **b**: PRF group, **c**: NB/PRF group, **d**: TDM/PRF group. Masson Goldner trichrome, x100

After one month, a light microscopic examination of the Masson Goldner trichrome-stained sections of the control group revealed the presence of numerous areas of unmineralized bone (stained red), (Fig. 9a). The PRF group showed few areas of unmineralized bone (Fig. 9b). NB/PRF and treated dentin matrix and PRF, showed scattered areas of unmineralized bone (Fig. 9c and d). After 3 months, the control group exhibited areas of unmineralized bone (Fig. 10a). All other treated groups showed marked improvement in bone mineralization, where homogenous mineralization of the bone matrix was noted (stained green) with minute areas of unmineralized bone (stained red) (Fig. 10b, c, and d).

### Histomorphometric analysis

#### Percentage of newly formed bone

After the first month, a statistically significant difference was noted between all studied groups (*p*_1_ < 0.001, *p*_2_ < 0.001, *p*_3_ = 0.042) regarding the percentage of newly formed bone, as shown in (Table 1). TDM/PRF showed the highest percentage of surface area of newly formed bone, followed by NB/PRF, PRF, and the control group where the values were (70.80 ± 2.95, 67.55 ± 3.07, 26.79 ± 2.55, and 10.71 ± 1.68), respectively. After 3 months, TDM/PRF showed the highest percentage, followed by NB/PRF, PRF, and the control group (84.60 ± 2.91, 78.81 ± 2.92, 52.99 ± 3.34, and 17.54 ± 2.21), respectively. There was also a statistically significant difference between all groups (*p*_*1*_ < 0.001,*p*_*2*_ < 0.001, *p*_*3*_ < 0.001). Moreover, all groups showed a statistically significant increase in bone surface area in the third month compared to the first month (*p* < 0.001).

**Table 1:** Comparison between the different studied groups according to percentage of bone surface area

Percentage of bone surface area	Control (*n* = 10)	PRF (*n* = 10)	NB/PRF (*n* = 10)	TDM/PRF (*n* = 10)	F	*P*
**First month**						
Min.– Max	7.82–13.25	22.19–30.63	63.03–72.03	65.96–74.77	1301.42^*^	< 0.001^*^
Mean ± SD.	10.71 ± 1.68	26.79^a^ ± 2.55	67.55^ab^ ± 3.07	70.80^abc^ ± 2.95		
***p***_**0**_		< 0.001^*^	< 0.001^*^	< 0.001^*^		
**Sig. bet. groups.**		*p*_1_ < 0.001^*^, *p*_2_ < 0.001^*^, *p*_3_ = 0.042^*^				
**Third month**						
Min.– Max	13.74–20.77	47.64–57.42	75.03–84.04	79.52–89.42	1132.73^*^	< 0.001^*^
Mean ± SD.	17.54 ± 2.21	52.99^a^ ± 3.34	78.81^ab^ ± 2.92	84.60^abc^ ± 2.91		
***p***_**0**_		< 0.001^*^	< 0.001^*^	< 0.001^*^		
**Sig. bet. groups.**		*p*_1_ < 0.001^*^, *p*_2_ < 0.001^*^, *p*_3_ < 0.001^*^				
***p***_**4**_	< 0.001^*^	< 0.001^*^	< 0.001^*^	< 0.001^*^		

**SD**: Standard deviation, **Min**: Minimum, **Max**: Maximum

**F**: **F for One way ANOVA test**, Pairwise comparison bet. each 2 groups was done using **Post Hoc Test (Tukey)**

p: p value for comparing between the studied groups

p_0_: p value for comparing between **Control** and each other group

p_1_: p value for comparing between **PRF** and **NB/PRF**

p_2_: p value for comparing between **PRF** and **TDM/PRF**

p_3_: p value for comparing between **NB/PRF** and **TDM/PRF**

p_4_: p value for **Student t-test for** comparing between **First month and Third month in each group**

*: Statistically significant at *p* ≤ 0.05

a: Significant with **Control**

b: Significant with **PRF**

c: Significant with **NB/PRF**

#### Percentage of unmineralized bone

Results of percentage of unmineralized bone are shown in (Table 2). After one month, the percentage of unmineralized bone was lowest in the PRF group, followed by TDM/PRF, NB/PRF, and control groups, where the values were (8.93 ± 2.60, 24.67 ± 3.55, 26.77 ± 2.78, and 28.90 ± 2.44), respectively. No significant difference was noted between TDM/PRF and NB/PRF (*p* = 0.375). Significant differences were noted between the PRF group and each of the NB /PRF and TDM/PRF (*p* < 0.001).

After 3 months, all groups showed a statistically significant decrease in the percentage of unmineralized bone (*p*_4_ < 0.001) compared to the first month. No statistically significant difference was noted between PRF and the TDM/PRF (*p* = 0.356). In addition, no significant difference was noted between NB/PRF and TDM/PRF (*p* = 0.352).

**Table 2:** Comparison between the different studied groups according to percentage of unmineralized bone

Percentage of unmineralized bone	Control (*n* = 10)	PRF (*n* = 10)	NB/PRF (*n* = 10)	TDM/PRF (*n* = 10)	F	*P*
**First month**						
Min.– Max	24.93–32.42	4.65–12.77	22.71–30.76	19.56–30.14	100.144^*^	< 0.001^*^
Mean ± SD.	28.90 ± 2.44	8.93^a^ ± 2.60	26.77^b^ ± 2.78	24.67^ab^ ± 3.55		
***p***_**0**_		< 0.001^*^	0.358	0.011^*^		
**Sig. bet. groups.**		*p*_1_ < 0.001^*^, *p*_2_ < 0.001^*^, *p*_3_ = 0.375				
**Third month**						
Min.– Max	5.65–14.32	0.39–2.31	0.99–6.32	0.84–5.33	51.239^*^	< 0.001^*^
Mean ± SD.	10.08 ± 2.52	1.28^a^ ± 0.70	3.86^ab^ ± 1.66	2.57^a^ ± 1.52		
***p***_**0**_		< 0.001^*^	< 0.001^*^	< 0.001^*^		
**Sig. bet. groups.**		*p*_1_ = 0.010^*^, *p*_2_ = 0.356, *p*_3_ = 0.352				
***p***_**4**_	< 0.001^*^	< 0.001^*^	< 0.001^*^	< 0.001^*^		

**SD**: Standard deviation, **Min**: Minimum, **Max**: Maximum

**F**: **F for One way ANOVA test**, Pairwise comparison bet. each 2 groups was done using **Post Hoc Test (Tukey)**

p: p value for comparing between the studied groups

p_0_: p value for comparing between **Control** and each other group

p_1_: p value for comparing between **PRF** and **NB/PRF**

p_2_: p value for comparing between **PRF** and **TDM/PRF**

p_3_: p value for comparing between **NB/PRF** and **TDM/PRF**

p_4_: p value for **Student t-test for** comparing between **First month and Third month in each group**

*: Statistically significant at *p* ≤ 0.05

a: Significant with **Control**

b: Significant with **PRF**

c: Significant with **NB/PRF**

### Immunohistochemical analysis

**Fig. 11:**
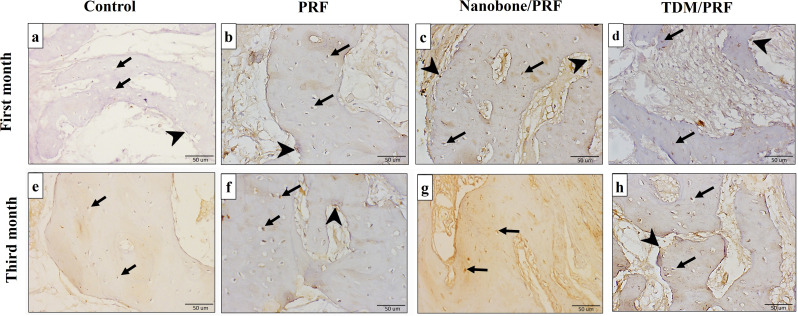
LM of osteopontin immune-expression in different experimental groups at 1 month (**a**-**d**) and third month (**e**-**h**). Control groups show weak reaction (**a**&**e**). Other groups show more intense osteopontin immune-expression in osteoblasts (arrow heads) and osteocytes (arrows)

Results of immune expression of osteopontin are shown in Fig. 11 and (Table 3). After one month, NB/PRF showed the highest osteopontin optical density followed by TDM/PRF and PRF where the values were 0.15, 0.11 & 0.09 respectively. The difference between NB/PRF and TDM/PRF was not significant (*p3* = 0.234). After 3 months, NB/PRF showed the highest osteopontin optical density followed by TDM/PRF and PRF where the values were 0.22, 0.19 & 0.14 respectively. The difference between NB/PRF and TDM/PRF was also not significant (*p3* = 0.607).

**Table 3:** Comparison between the different studied groups according to optical density of osteopontin

Optical density of osteopontin	Control (*n* = 10)	PRF (*n* = 10)	NB/PRF (*n* = 10)	TDM/PRF (*n* = 10)	F	*p*
**First month**						
Min.– Max	0.01–0.11	0.03–0.21	0.06–0.27	0.05–0.17	6.346^*^	0.001^*^
Mean ± SD.	0.06 ± 0.03	0.09 ± 0.05	0.15^ab^ ± 0.06	0.11 ± 0.04		
**p**_**0**_		0.393	0.001^*^	0.103		
**Sig. bet. grps.**		p_1_ = 0.049^*^, p_2_ = 0.870, p_3_ = 0.234				
**Third month**						
Min.– Max	0.01–0.17	0.08–0.22	0.07–0.32	0.12–0.25	9.785^*^	< 0.001^*^
Mean ± SD.	0.09 ± 0.05	0.14 ± 0.05	0.22^ab^ ± 0.08	0.19^a^ ± 0.04		
**p**_**0**_		0.260	< 0.001^*^	0.003^*^		
**Sig. bet. grps.**		p_1_ = 0.016^*^p_2_ = 0.232, p_3_ = 0.607				
**p**_**4**_	0.074	0.018^*^	0.088	< 0.001^*^		

**SD**: Standard deviation, **Min**: Minimum, **Max**: Maximum

**F**: **F for One way ANOVA test**, Pairwise comparison bet. each 2 groups was done using **Post Hoc Test (Tukey)**

p: p value for comparing between the studied groups

p_0_: p value for comparing between **Control** and each other groups

p_1_: p value for comparing between **PRF** and **NB/PRF**

p_2_: p value for comparing between **PRF** and **TDM/PRF**

p_3_: p value for comparing between **NB/PRF** and **TDM/PRF**

p_4_: p value for **Student t-test for** comparing between **First month and Third month** in each group

*: Statistically significant at *p* ≤ 0.05

a: Significant with **Control**

b: Significant with **PRF**

c: Significant with **NB/PRF**

## Discussion

The aim of this study was to evaluate the bone forming ability of combining TDM with PRF as a socket preservation material immediately following tooth extraction in comparison to synthetic bone grafting material (NB) combined with PRF. The null hypothesis was rejected as results revealed an increase in surface area of bone formation by scaffolds made of TDM/PRF compared to NB/PRF when used as socket preservation materials.

TDM has proven its ability in cell adhesion due to its hydrophilic surface and odontogenic and osteogenic potential due to the presence of collagenous and non-collagenous proteins and growth factors, thus having osteoconductive and osteoinductive potential [39]. NB is a synthetic bone-grafting substitute made of nano-hydroxyapatite and nanoporous silica (SiO_2_), characterized by its high porosity and osteoconductivity [40].

In vitro characterization of both scaffolds using FTIR revealed characteristic groups of TDM/PRF, NB /PRF, which are similar to the findings of Guo et al. [41] and Sadlo et al. [42], respectively. SEM images of both scaffolds revealed a porous and mesh-like structure of PRF, which is essential for angiogenesis and bone formation [43].

Histological analysis revealed that the PRF group showed formation of new thick cancellous bone trabeculae and small osteons. Histomorphometric analysis revealed a significant increase in new bone surface area in the PRF group compared to the control group after both the first and third months (*p* < 0.001). Our results are in agreement with previous studies that found a positive effect of PRF on the enhancement of new bone formation in extraction sockets [18, 27]. PRF can stimulate bone formation as it contains several growth factors that can upregulate the differentiation of osteogenic cells and thus increase bone regeneration [44]. PRF can increase the expression of osteogenic genes like Runt-related transcription factor 2 (Runx2), and osterix (OSX) [45].

Histological examination using Masson trichrome-stained sections in the PRF group revealed homogenous mineralization of the newly formed bone. Histomorphometric analysis showed that PRF exhibited the lowest percentage of unmineralized bone among all groups in both intervals (8.93 ± 2.6 and 1.28 ± 0.7, respectively). A review article by Liu et al. stated that from in vitro studies, PRF can increase osteogenic cell migration, proliferation, and mineralization. However, from clinical studies, PRF alone may be not sufficient, and it should be mixed with other bone forming materials or drugs [46].

Histological results of this study revealed higher bone formation in the NB/PRF group compared to both the control and PRF groups. More cancellous bone trabeculae were noticed surrounding well-vascularized bone marrow tissue. Histomorphometric analysis revealed a significant increase in new bone formation in the NB/PRF group compared to the control and PRF groups at both the first and third months (*p* < 0.001). A previous study by Jaber et al. also affirmed the positive effect of NB on bone formation. They found a significant increase in new bone formation in the NB group compared to the control group [47]. Another clinical study concluded that the combination of NB and PRF resulted in enhancement of bone formation and density in bone defects after mandibular cyst enucleation [48].

NB can increase new bone formation because its structure at the nanoscale level mimics the natural bone topography. This nanostructure can induce osteogenic cell attachment, differentiation, and new vascularization. It can act as a biomimetic niche for the induction of bone regeneration [49]. Histomorphometric analysis revealed a significant decrease in the percentage of unmineralized bone in NB compared to the control group at the third month (*p* < 0.001). This can be attributed to the effect of NB on bone mineralization. Hakam et al. [50] found that NB increases the expression of Runx-2, which is a main factor for bone matrix formation and mineralization [51].

Histomorphometric results revealed that there was a significant increase in the new bone formation percentage of in TDM/PRF in comparison to the control group at both first and third months (*p* < 0.001). Moreover, there was a significant increase in the percentage of newly formed bone in TDM/PRF compared to the NB/PRF group at both intervals (*p* = 0.042 and < 0.001 in the first and third months, respectively). Obvious bone formation in the TDM group was noted around the remnants of the dentin matrix. These results are in agreement with those of Reis et al. [52], who found that human demineralized dentin matrix can significantly increase new bone formation in rats tooth sockets compared to the blood clot group. In addition, Moraes et al. [53] investigated the effect of human demineralized dentin matrix (HDDM), xenograft, and autogenous bone in rat socket preservation, and concluded that HDDM can be a biocompatible material to minimize post-extraction bone loss volume.

TDM has different properties which enhance bone formation. It preserves the structure of dentinal tubules, which act as a scaffold for cells and nutrients [24]. It also contains different growth factors that enhance stem cell proliferation and differentiation to enhance alveolar bone formation [54]. In addition, Sultan et al. found that TDM upregulates collagen I expression and increases activity of alkaline phosphatase in bone marrow mesenchymal stem cells, thus increasing bone formation [55].

The results of the Goldner-Masson trichrome-stained sections revealed a significant decrease in the percentage of unmineralized bone in the TDM group in comparison to the control group in both the first and third months (*p* = 0.011 and < 0.001, respectively). Moreover, no significant differences were seen between TDM/PRF and NB/PRF groups at both intervals (*p* = 0.375 and 0.352, respectively). This can be attributed to the effect of TDM on bone mineralization. Luo et al. [56] found that TDM can significantly increase the expression of mineralization-related markers, including alkaline phosphatase, (RUNX2), and osteocalcin, in jaw bone marrow mesenchymal stem cells.

Osteopontin is a cell matrix protein that has an important role in bone metabolism and regulation of different cellular activities of bone cells [57]. The immune-histochemical analysis of the current study revealed an increase in optical density of osteopontin expression in NB/PRF group & TDM/PRF. Thieu et al. stated that the increase in osteopontin expression may indicate future bone growth because it is mandatory for new bone formation [58]. It enhances mesenchymal stem cells proliferation and migration to resorbed bone surface [59]. Osteopontin is also important in binding osteoblast cells to bone surface and good integration of new bone to the old bone at the periphery of the socket area [60]. In addition, it was suggested that it plays an important role in bone mineralization [61].

A recent study by Ouyang et al. concluded that autologous partially demineralized dentin matrix can achieve comparable results to deproteinized bovine bone mineral in bone augmentation in alveolar bone deficiency in orthodontic patients [62]. Further studies are still needed to fully examine the complete bone formation after more than three months, using other bigger animal species, such as dogs, and comparing the TDM/PRF mixture with more than one synthetic bone grafting material. Further studies could be done to confirm the clinical success of TDM/PRF for socket preservation following extraction after optimization of the manufacturing parameters to be a potential autologous material for socket preservation.

## Conclusions

TDM/PRF showed the ability to form new bone in extraction sockets in rabbits. This effect can be attributed to its osteoinductive and osteoconductive properties and increased expression of osteopontin.

## Electronic supplementary material

Below is the link to the electronic supplementary material.

Supplementary Material 1

Supplementary Material 2

## Data Availability

All data generated or analyzed during this study are included in this published article.

## Abbreviations

FTIR: Fourier Transform Infrared Spectroscopy
H & E: Hematoxylin and eosin
PRF: Platelet-rich fibrin
SEM: Scanning electron microscope
TDM: Treated dentine matrix
NB: Nanobone
