# Supplementary material for: Histomorphometric and immunohistochemical assessment of treated dentin matrix delivered by platelet-rich fibrin for socket preservation in rabbit model
Source: BMC Oral Health. 2025 Feb 12;25:225. doi: 10.1186/s12903-025-05569-3 (PMC11823050; doi:10.1186/s12903-025-05569-3)
Supplement: Supplementary file 1 — Supplementary Material 1 [file 12903_2025_5569_MOESM1_ESM.docx]

**Raw Data File**

**Percentage of bone surface area**

|  | **One-month groups** | | | |
| --- | --- | --- | --- | --- |
|  | **Control** | **PRF** | **Nanobone /PRF** | **TDM/PRF** |
| **1** | 10.7041053 | 26.75701 | 67.53068811 | 70.82698371 |
| **2** | 13.245912 | 24.16278 | 66.04272 | 68.64747 |
| **3** | 8.8082325 | 22.19375 | 63.02626 | 66.853647 |
| **4** | 11.887132 | 28.28217 | 70.42526 | 72.63738 |
| **5** | 9.592383 | 29.28271 | 72.02713 | 73.73636 |
| **6** | 12.1299838 | 25.13712 | 64.28374 | 65.96373 |
| **7** | 7.821373 | 27.21334 | 64.26267 | 69.74637 |
| **8** | 10.213898 | 28.52373 | 67.84748 | 71.86254 |
| **9** | 10.582623 | 25.68787 | 70.03176 | 74.76549 |
| **10** | 12.1233 | 30.62662 | 70.04744 | 72.96543 |
| **mean** | 10.7108943 | 26.78671 | 67.55251481 | 70.80054007 |
|  | **Three-months groups** | | | |
|  | **Control** | **PRF** | **Nanobone /PRF** | **TDM/PRF** |
| **1** | 17.3222 | 52.97782 | 78.70219938 | 84.7620517 |
| **2** | 17.03736 | 56.65423 | 76.26634 | 86.76421 |
| **3** | 20.77377 | 57.42325 | 75.02635 | 89.42323 |
| **4** | 15.73735 | 53.62412 | 79.01365 | 81.61252 |
| **5** | 13.73738 | 49.63535 | 82.76358 | 79.52432 |
| **6** | 20.76154 | 53.64234 | 77.73736 | 85.52142 |
| **7** | 18.53637 | 55.72663 | 75.73738 | 87.51242 |
| **8** | 16.76543 | 48.73635 | 84.03634 | 83.42324 |
| **9** | 16.07543 | 47.63638 | 80.31735 | 82.94242 |
| **10** | 18.65413 | 53.86353 | 78.53424 | 84.54242 |
| **mean** | 17.540096 | 52.992 | 78.81347894 | 84.60282517 |

**Percentage of unmineralized bone**

|  | **One-month groups** | | | |
| --- | --- | --- | --- | --- |
|  | **Control** | **PRF** | **Nanobone /PRF** | **TDM/PRF** |
| **1** | 28.942 | 8.952 | 26.716 | 24.661 |
| **2** | 30.976 | 9.543 | 28.825 | 25.765 |
| **3** | 31.765 | 11.876 | 29.876 | 29.432 |
| **4** | 27.876 | 12.765 | 30.764 | 30.143 |
| **5** | 26.654 | 10.871 | 27.982 | 27.087 |
| **6** | 29.962 | 7.876 | 25.752 | 20.234 |
| **7** | 32.423 | 6.897 | 23.524 | 22.054 |
| **8** | 26.543 | 5.951 | 23.765 | 23.765 |
| **9** | 24.927 | 4.654 | 22.712 | 19.564 |
| **10** | 28.964 | 9.887 | 27.765 | 24.021 |
| **mean** | 28.9032 | 8.9272 | 26.7681 | 24.6726 |
|  | **Three-months groups** | | | |
|  | **Control** | **PRF** | **Nanobone /PRF** | **TDM/PRF** |
| **1** | 10.025 | 1.293 | 3.848 | 2.569 |
| **2** | 11.082 | 2.265 | 2.543 | 3.365 |
| **3** | 14.321 | 1.042 | 5.453 | 4.321 |
| **4** | 12.756 | 1.524 | 1.954 | 5.332 |
| **5** | 11.436 | 2.312 | 0.988 | 3.432 |
| **6** | 9.542 | 0.952 | 4.765 | 1.654 |
| **7** | 7.549 | 1.876 | 5.123 | 1.145 |
| **8** | 5.651 | 0.632 | 3.943 | 0.976 |
| **9** | 8.432 | 0.543 | 6.321 | 0.843 |
| **10** | 9.998 | 0.393 | 3.623 | 2.034 |
| **mean** | 10.0792 | 1.2832 | 3.8561 | 2.5671 |

**Osteopontin Optical Density**

|  | **One-month groups** | | | |
| --- | --- | --- | --- | --- |
|  | **Control** | **PRF** | **Nanobone /PRF** | **TDM/PRF** |
| **1** | 0.058235317 | 0.090569835 | 0.134698574 | 0.106379643 |
| **2** | 0.1008765 | 0.07768 | 0.161445 | 0.143536 |
| **3** | 0.0376352 | 0.10013 | 0.198474 | 0.128724 |
| **4** | 0.0463637 | 0.02534 | 0.273646 | 0.1172535 |
| **5** | 0.0694743 | 0.05737 | 0.123245 | 0.0653436 |
| **6** | 0.0112348 | 0.04243 | 0.115464 | 0.056337 |
| **7** | 0.0752342 | 0.08053 | 0.1087353 | 0.173635 |
| **8** | 0.105232 | 0.20747 | 0.132113 | 0.0474746 |
| **9** | 0.0542326 | 0.10988 | 0.156427 | 0.098283 |
| **10** | 0.016534 | 0.11332 | 0.0564535 | 0.1253536 |
| **mean** | 0.057505262 | 0.090471983 | 0.146070137 | 0.106231994 |
|  | **Three-months groups** | | | |
|  | **Control** | **PRF** | **Nanobone /PRF** | **TDM/PRF** |
| **1** | 0.105510185 | 0.137027236 | 0.236278465 | 0.194352576 |
| **2** | 0.15134 | 0.116373 | 0.072365 | 0.211321 |
| **3** | 0.16653 | 0.093635 | 0.286153 | 0.253425 |
| **4** | 0.08735 | 0.153456 | 0.117643 | 0.190718 |
| **5** | 0.06543 | 0.1198736 | 0.172324 | 0.181432 |
| **6** | 0.01008 | 0.082736 | 0.265431 | 0.121113 |
| **7** | 0.11423 | 0.076445 | 0.204332 | 0.219765 |
| **8** | 0.06543 | 0.175674 | 0.1954342 | 0.156743 |
| **9** | 0.03242 | 0.220009 | 0.321544 | 0.165543 |
| **10** | 0.10321 | 0.200876 | 0.311123 | 0.173425 |
| **mean** | 0.090153018 | 0.137610484 | 0.218262767 | 0.186783758 |
