# Supplementary material for: Histomorphometric and immunohistochemical assessment of treated dentin matrix delivered by platelet-rich fibrin for socket preservation in rabbit model
Source: BMC Oral Health. 2025 Feb 12;25:225. doi: 10.1186/s12903-025-05569-3 (PMC11823050; doi:10.1186/s12903-025-05569-3)
Supplement: Supplementary file 2 — Supplementary Material 2 [file 12903_2025_5569_MOESM2_ESM.docx]

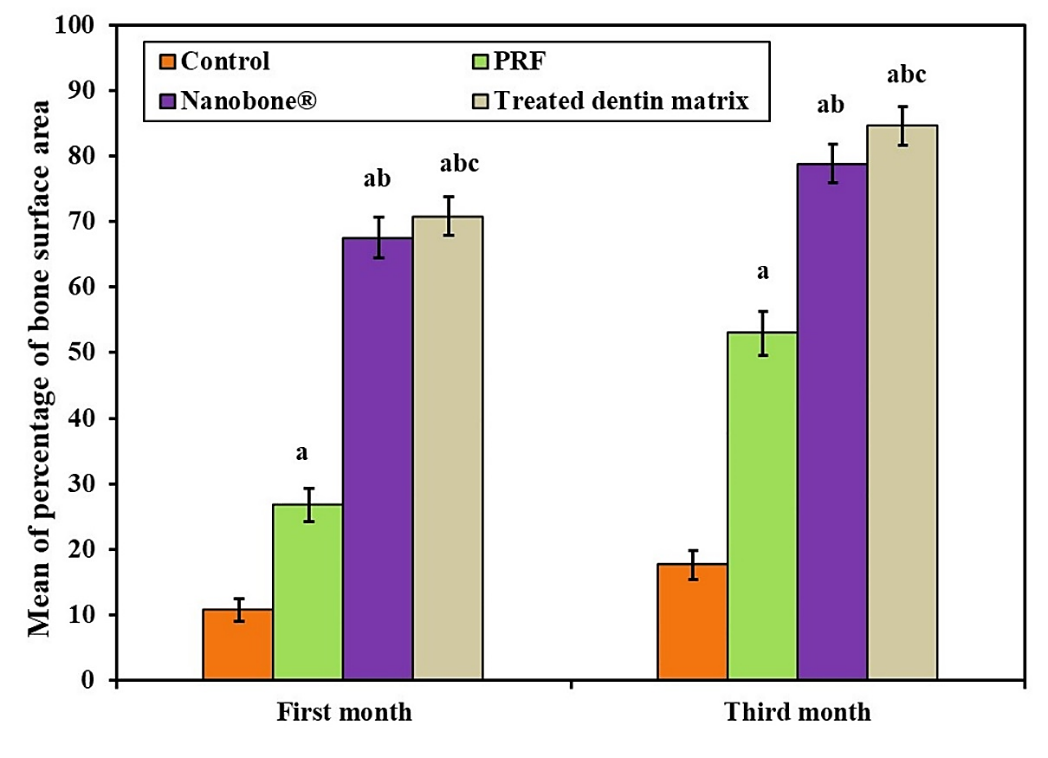


**S1:** Comparison between the different studied groups according to percentage of bone surface area

(a: Significant with **Control,** b: Significant with **PRF**, c: Significant with **NB/PRF)**


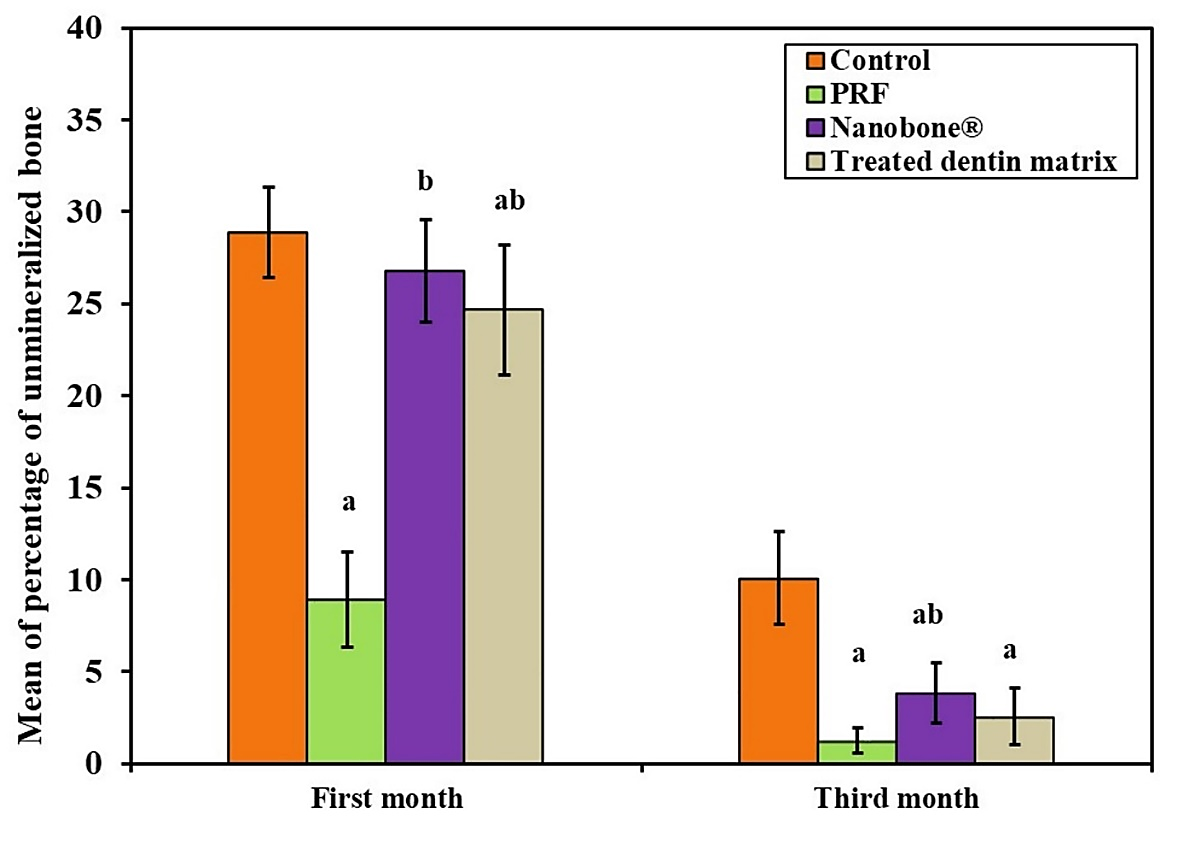


**S2:** Comparison between the different studied groups according to percentage of unmineralized bone (a: Significant with **Control,** b: Significant with **PRF**, c: Significant with **NB/PRF)**


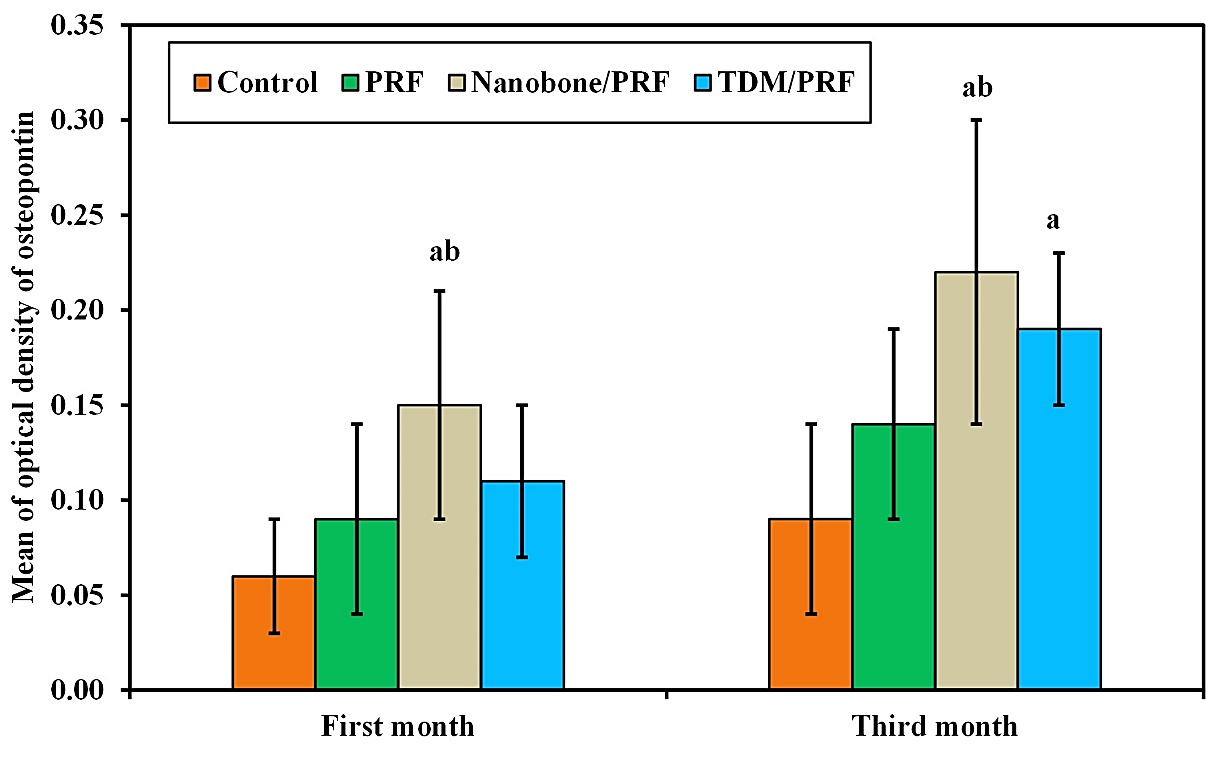


**S3:** Comparison between the different studied groups according to optical density of osteopontin
